# New Genomics Discoveries Across the Bipolar Disorder Spectrum Implicate Neurobiological and Developmental Pathways

**DOI:** 10.1016/j.biopsych.2025.05.020

**Published:** 2025-05-31

**Authors:** Kevin S. O’Connell, Rolf Adolfsson, Till F.M. Andlauer, Michael Bauer, Bernhaud Baune, Joanna M. Biernacka, Bernardo Carpiniello, Sven Cichon, Nick Craddock, Alfredo B. Cuellar-Barboza, Udo Dannlowski, Franziska Degenhardt, Dimitris Dikeos, Panagiotis Ferentinos, Andreas J. Forstner, Mark A. Frye, Janice M. Fullerton, Maria Grigoroiu-Serbanescu, José Guzman-Parra, Lisa Jones, Lina Jonsson, Tilo Kircher, Manolis Kogevinas, Mikael Landén, Jolanta Lissowska, Christine Lochner, Mirko Manchia, Fermin Mayoral, Susan L. McElroy, Nathaniel W. McGregor, Andreas Meyer-Lindenberg, Philip B. Mitchell, Bertram Müller-Myhsok, Markus M. Nöthen, George P. Patrinos, Joanna M. Pawlak, Andreas Reif, Marcella Rietschel, Peter R. Schofield, Thomas G. Schulze, Alessandro Serretti, Jordan W. Smoller, Alessio Squassina, Dan J. Stein, Fabian Streit, Beata Świątkowska, Leonardo Tondo, Eduard Vieta, Irwin D. Waldman, Anders M. Dale, Ole A. Andreassen

**Affiliations:** Center for Precision Psychiatry, Division of Mental Health and Addiction, Oslo University Hospital, and Institute of Clinical Medicine, University of Oslo, Oslo, Norway (KSO, OAA); Department of Clinical Sciences, Psychiatry, Umeå University Medical Faculty, Umeå, Sweden (RA); Department of Neurology, Klinikum rechts der Isar, School of Medicine, Technical University of Munich, Munich, Germany (TFMA); Department of Psychiatry and Psychotherapy, University Hospital Carl Gustav Carus, Technische Universität Dresden, Dresden, Germany (MB); Department of Psychiatry, University of Münster, Münster, Germany (BB); Department of Psychiatry, University of Melbourne, Melbourne, Victoria, Australia (BB); Florey Institute of Neuroscience and Mental Health, Parkville, Victoria, Australia (BB); Department of Quantitative Health Sciences, Mayo Clinic, Rochester, Minnesota (JMB); Department of Psychiatry and Psychology, Mayo Clinic, Rochester, Minnesota (JMB, MAF); Unit of Psychiatry, Department of Medical Sciences and Public Health, University of Cagliari, Cagliari, Italy (BC, MM); Unit of Clinical Psychiatry, Department of Medicine, University Hospital Agency of Cagliari, Cagliari, Italy (BC, MM); Department of Biomedicine, University of Basel, Basel, Switzerland (SC); Institute of Human Genetics, University of Bonn, School of Medicine & University Hospital Bonn, Bonn, Germany (SC); Institute of Medical Genetics and Pathology, University Hospital Basel, Basel, Switzerland (SC); Institute of Neuroscience and Medicine, Research Centre Jülich, Jülich, Germany (SC, AJF); National Centre for Mental Health, Cardiff University, Cardiff, United Kingdom (NC); Department of Psychiatry, Universidad Autónoma de Nuevo León, Monterrey, Mexico (ABC-B); Department of Psychiatry and Psychology, Mayo Clinic, Rochester, Minnesota (ABC-B); Institute for Translational Psychiatry, University of Münster, Münster, Germany (UD); Institute of Human Genetics, University of Bonn, School of Medicine & University Hospital Bonn, Bonn, Germany (FD, AJF, MMN); Department of Child and Adolescent Psychiatry, Psychosomatics and Psychotherapy, University Hospital Essen, University of Duisburg-Essen, Duisburg, Germany (FD); National and Kapodistrian University of Athens, 1st Department of Psychiatry, Eginition Hospital, Athens, Greece (DD); National and Kapodistrian University of Athens, 2nd Department of Psychiatry, Attikon General Hospital, Athens, Greece (PF); Social, Genetic and Developmental Psychiatry Centre, King’s College London, London, United Kingdom (PF); Department of Psychiatry, Marburg University, Marburg, Germany (AJF, TK); Neuroscience Research Australia, Sydney, New South Wales, Australia (JMF); School of Biomedical Sciences, Faculty of Medicine & Health, University of New South Wales, Sydney, New South Wales, Australia (JMF); Psychiatric Genetics Research Unit, Alexandru Obregia Clinical Psychiatric Hospital, Bucharest, Romania (MG-S); Mental Health Department, University Regional Hospital, Biomedicine Institute, Málaga, Spain (JG-P, FM); Three Counties Medical School, University of Worcester, Worcester, United Kingdom (LJone); Institute of Neuroscience and Physiology, University of Gothenburg, Gothenburg, Sweden (LJons, ML); ISGlobal, Barcelona, Spain (MK); Department of Medical Epidemiology and Biostatistics, Karolinska Institutet, Stockholm, Sweden (ML); Cancer Epidemiology and Prevention, Maria Sklodowska-Curie National Research Institute of Oncology, Warsaw, Poland (JL); South African Medical Research Council Unit on Risk & Resilience in Mental Disorders, Department of Psychiatry, Stellenbosch University, Stellenbosch, South Africa (CL); Department of Pharmacology, Dalhousie University, Halifax, Nova Scotia, Canada (MM); Research Institute, Lindner Center of HOPE, Mason, Ohio (SLM); Human and Systems Genetics Working Group, Department of Genetics, Stellenbosch University, Stellenbosch, South Africa (NWM); Department of Psychiatry and Psychotherapy, Central Institute of Mental Health, Medical Faculty Mannheim, University of Heidelberg, Mannheim, Germany (AML, FS); German Centre for Mental Health, Germany (AM-L); Discipline of Psychiatry and Mental Health, School of Clinical Medicine, Faculty of Medicine & Health, University of New South Wales, Sydney, New South Wales, Australia (PBM, PRS); Department of Translational Research in Psychiatry, Max Planck Institute of Psychiatry, Munich, Germany (BM-M); Munich Cluster for Systems Neurology, Munich, Germany (BM-M); University of Liverpool, Liverpool, United Kingdom (BM-M); University of Patras, School of Health Sciences, Department of Pharmacy, Laboratory of Pharmacogenomics and Individualized Therapy, Patras, Greece (GPP); United Arab Emirates University, College of Medicine and Health Sciences, Department of Genetics and Genomics, Al-Ain, Abu Dhabi, United Arab Emirates (GPP); United Arab Emirates University, Zayed Center for Health Sciences, Al-Ain, Abu Dhabi, United Arab Emirates (GPP); Erasmus University Medical Center, Faculty of Medicine and Health Sciences, Department of Pathology, Clinical Bioinformatics unit, Rotterdam, the Netherlands (GPP); Department of Psychiatry, Department of Psychiatric Genetics, Poznan University of Medical Sciences, Poznań, Poland (JMP); Department of Psychiatry, Psychosomatic Medicine and Psychotherapy, University Hospital Frankfurt, Frankfurt am Main, Germany (AR); Fraunhofer Institute for Translational Medicine and Pharmacology, Frankfurt am Main, Germany (AR, FS); Department of Psychiatry and Behavioral Sciences, Norton College of Medicine, State University of New York Upstate Medical University, Syracuse, New York (MR, TGS); Institute of Psychiatric Phenomics and Genomics, Ludwig Maximilian University of Munich University Hospital, Ludwig Maximilian University of Munich, Munich, Germany (TGS); Department of Psychiatry and Behavioral Sciences, Johns Hopkins University School of Medicine, Baltimore, Maryland (TGS); Department of Medicine and Surgery, Kore University of Enna, Enna, Italy (AS); Oasi Research Institute, Istituto di Ricovero e Cura a Carattere Scientifico, Troina, Italy (AS); Psychiatric and Neurodevelopmental Genetics Unit, Center for Genomic Medicine, Massachusetts General Hospital, Boston, Massachusetts (JWS); Center for Precision Psychiatry, Department of Psychiatry, Massachusetts General Hospital, Boston, Massachusetts (JWS); Department of Biomedical Sciences, University of Cagliari, Cagliari, Italy (AS); South African Medical Research Council Unit on Risk & Resilience in Mental Disorders, Department of Psychiatry & Neuroscience Institute, University of Cape Town, Cape Town, South Africa (DJS); Hector Institute for Artificial Intelligence in Psychiatry, Central Institute of Mental Health, Medical Faculty Mannheim, Heidelberg University, Mannheim, Germany (FS); German Center for Mental Health, Partner Site Mannheim-Heidelberg-Ulm, Germany (FS); Department of Environmental Epidemiology, Nofer Institute of Occupational Medicine, Łódź, Poland (BŚ); Lucio Bini Mood Disorder Center, Cagliari, Italy (LT); Department of Psychiatry, Harvard Medical School, Boston, Massachusetts (LT); Institute of Neuroscience, University of Barcelona, Barcelona, Spain (EV); Hospital Clínic, Institut d’Investigacions Biomèdiques August Pi i Sunyer, Barcelona, Spain (EV); CIBERSAM, Barcelona, Spain (EV); Department of Psychology, Emory University, Atlanta, Georgia (IDW); Multimodal Imaging Laboratory, University of California San Diego, La Jolla, California (AMD); Center for Multimodal Imaging and Genetics, J. Craig Venter Institute, La Jolla, California (AMD); and KG Jebsen Centre for Neurodevelopmental Disorders, University of Oslo and Oslo University Hospital, Oslo, Norway (OAA).

## Abstract

Bipolar disorder (BD) is a highly heritable mental disorder that affects millions of people worldwide. Our understanding of the genetic etiology and biological processes that underlie BD have greatly increased in recent years. Extensive progress has been made in identifying common variant signals for BD, and the polygenic score from the latest genome-wide association study (GWAS) may provide some clinical utility if combined with other risk factors for BD. The role of rare variation in BD remains to be determined, although genes annotated to common variant loci are shown to be enriched for rare variation. BD subtypes have been shown to differ in their genetic architecture, and as such, genetic studies across the subtypes of the BD spectrum will identify subtype-specific signals and reveal subtype-specific biological mechanisms. Despite this, subtype-specific GWAS sample sizes have not increased at the same rate as BD cases, and more concerted efforts are required to obtain this information for participants included in future BD GWASs. Moreover, assessment of culture, geography, and other systematic differences that may impact patient assessment will be necessary to ensure accurate inclusion of diverse ancestral groups and global representation in genetic studies of BD moving forward.

Bipolar disorder (BD) is a complex, often chronic psychiatric disorder that results in reduced functional ability and impaired quality of life (1). A core characteristic of BD is the episodic nature, with periods of elevation and depression of mood and behavior separated by periods of euthymia (2). The severity of the mood episodes differentiates the 2 main subtypes of BD. Bipolar I disorder (BDI) is characterized by episodes of mania and depression, whereas bipolar II disorder (BDII) includes episodes of hypomania and depression. While the lifetime prevalence is estimated at approximately 1% for each of these BD subtypes (3,4), large ranges have been reported (BDI: 0.1%–1.7%, BDII: 0.1%–3.0%) (3–5).

Family, twin, and adoption studies suggest that BD is heritable, with lifetime prevalence rates of BD between 5% and 10% in first-degree relatives of patients and between 40% and 70% in monozygotic twins (6). The heritability of BD is estimated to be between 60% and 85% based on twin studies (7,8), highlighting the importance of genetic variation in its etiology. As such, elucidating the genetic architecture of BD is crucial to understanding the underlying molecular mechanisms that influence the disorder. In this review, we outline the progress in genetic studies of BD and highlight novel insights into the genetic architecture across the BD subtypes and neurobiological mechanisms of the disorder.

## COMMON VARIATION

### Discovery

The first published large-scale genome-wide association study (GWAS) of BD included 2000 cases and 3000 controls and only reported 1 independent association signal at *p* < 5 × 10^−7^ (9). Subsequently, numerous other GWASs were published, with few significant findings due to limited sample size (10). This changed with the initiation of the Psychiatric Genomics Consortium (PGC), combining and meta-analyzing data across BD cohorts globally. The significantly larger and more well-powered GWASs of BD paved the way for a series of discoveries (11,12). Despite major gains in common variant discovery, most cohorts included in these studies comprised individuals of European ancestry, limiting the generalizability of the results (13,14).

**Figure 1.**
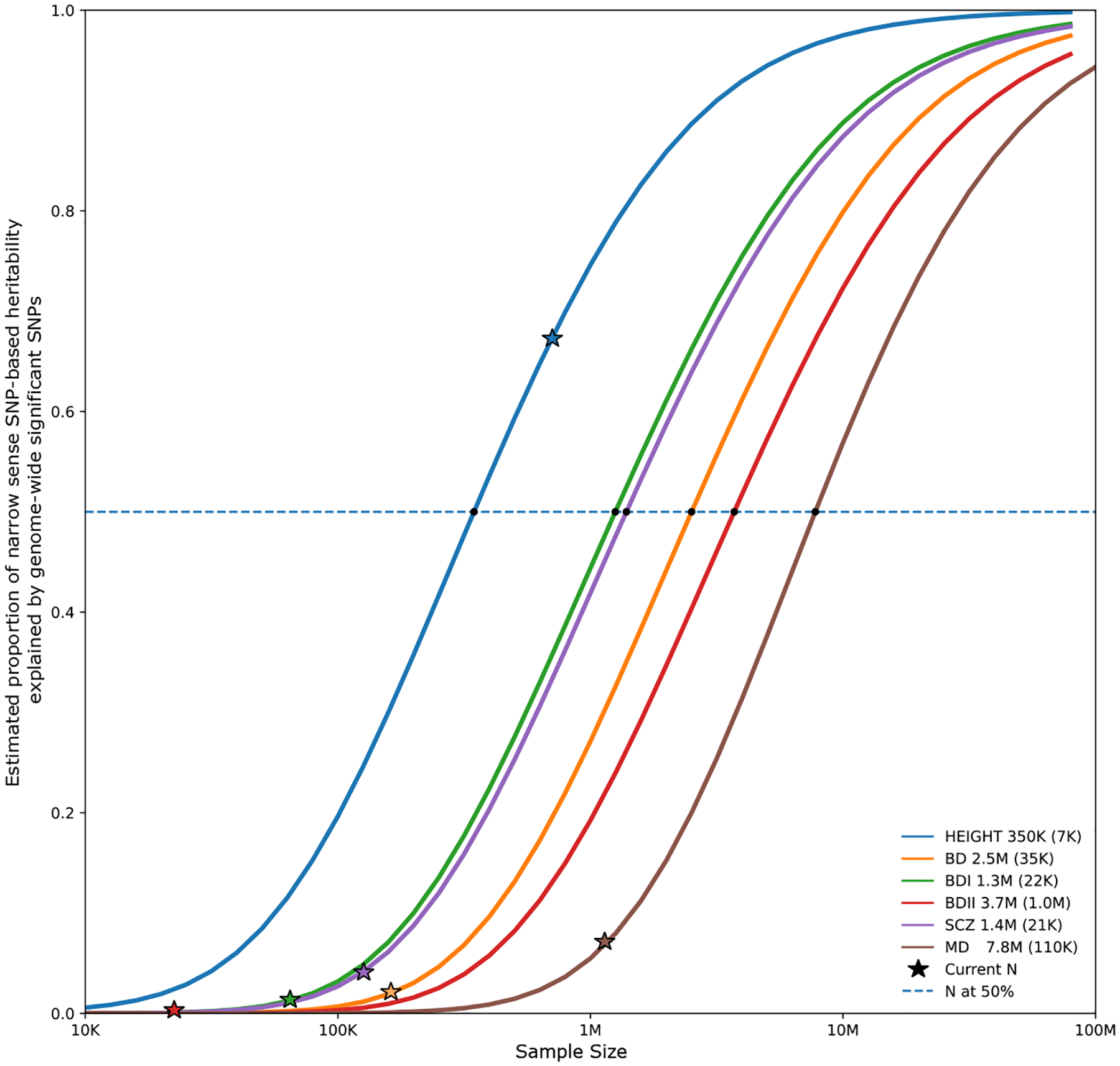
Progress in discovery from GWASs of BD (15), SCZ (18), and MD (60). The proportion of narrow-sense SNP-based heritability captured by the detected variants with genome-wide significance (vertical axis) can be calculated for increasing GWAS effective sample sizes (*N*, horizontal axis) using the univariate causal mixture model (27). The estimated GWAS effective sample sizes needed to capture 50% of the genetic variance (horizontal dashed line) associated with each disorder are marked with circles and listed in the plot legend with the estimated standard errors in parentheses. Stars indicate the effective sample sizes of analyzed GWASs. Height (61) is included as a comparator phenotype for which a large proportion of the additive SNP-based heritability is explained by genome-wide significant SNPs. The estimates shown here do not account for other forms of genetic variation, such as copy number variants, short tandem repeats, or rare variants. BD, bipolar disorder; BDI, bipolar I disorder; BDII, bipolar II disorder; GWAS, genome-wide association study; MD, major depression; SCZ, schizophrenia; SNP, single nucleotide polymorphism.

However, the most recent PGC study included more than 158,000 BD cases from 78 cohorts of European, African American, East Asian, and Latino ancestry. A total of 298 genome-wide significant loci were associated with the disorder (15) (Figure 1). These results suggest that an inflection point, where significant associations accumulate with smaller increases in sample size, has been reached for GWASs of BD and that inclusion of more samples will drastically increase genetic findings. Nevertheless, the rate of discovery in BD is considerably lower than in schizophrenia (SCZ), which may be due to clinical and genetic heterogeneity of BD (12,15) (Figure 1). As shown, the discoverability of BDI is much greater than that of BDII, with the latter requiring larger sample sizes to achieve similar discoverability.

### Neurobiological and Developmental Insights

**Figure 2.**
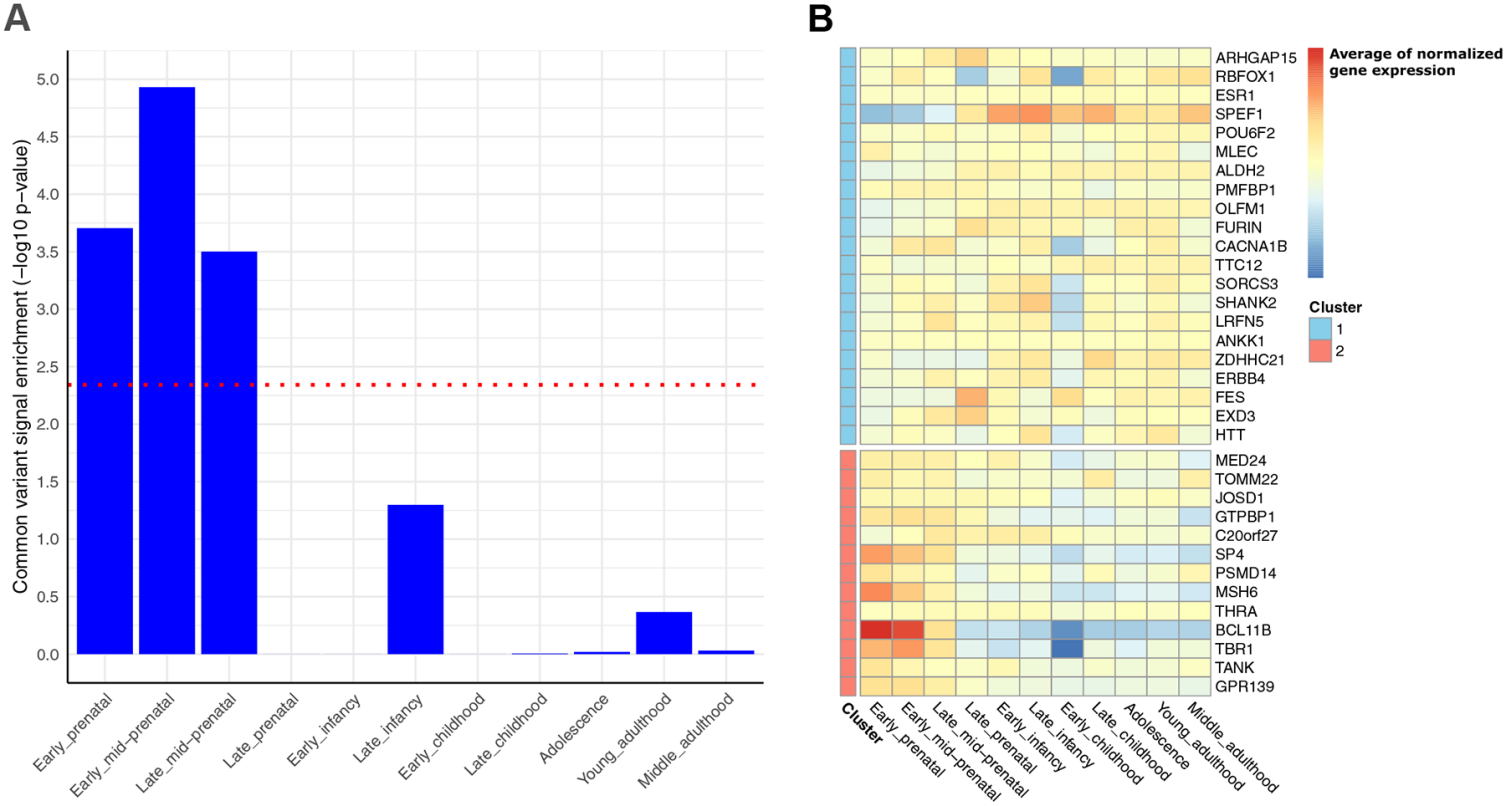
**(A)** Enrichment of the common genetic variant signal from the latest BD GWAS in brain samples across human developmental stages. BD GWAS signal was enriched in prenatal stages of development. Gene-set associations were performed using a single nucleotide polymorphism–wise mean model (±10-kb window) implemented in MAGMA (15,62). The dotted red line indicates the Bonferroni-corrected significance threshold. **(B)** Normalized gene expression of credible BD-associated genes, mapped to genome-wide significant loci in the latest BD GWAS, in brain samples across human developmental stages. These genes were further clustered into 2 distinct clusters based on their temporal expression patterns, with cluster 1 showing lower expression prenatally and cluster 2 showing higher expression prenatally with lower expression after birth. BD, bipolar disorder; GWAS, genome-wide association study.

BD GWAS signals are consistently enriched for genes expressed in brain tissue and, more specifically, in gene sets related to the synapse and transcription factor activity (12,15). Moreover, recent analyses highlighted that this enrichment is specific to the early- to mid-prenatal stages of development (15) (Figure 2A). Investigation of BD GWAS signals in smaller, more biologically relevant gene sets, utilizing a novel gene-set analysis tool (16), showed enrichment of numerous dopamine- and calcium-related biological processes driven by the *DRD2* and *CACNA1B* genes, respectively, as well as development of GABAergic (gamma-aminobutyric acidergic) interneurons (15). These results are consistent with the dopamine hypothesis of BD (17) and corroborate previous findings that implicate calcium channel activity in the disorder (12). These findings provide drug repurposing opportunities for the treatment of BD.

More specific analyses at the single-cell level implicate neuronal populations from different brain regions, including hippocampal pyramidal neurons and interneurons of the prefrontal cortex and hippocampus (12,15). These findings are consistent across BD GWAS iterations (12,15) and are similar to observations in SCZ (18). Similar patterns are also observed across ascertainment and BD subtypes (15). Furthermore, enrichment analyses of BD, SCZ, and depression in single-nucleus RNA sequencing (RNA-seq) data of 3.369 million nuclei from 106 anatomical dissections within 10 brain regions highlight disorder-specific enrichment (15,19). For BD, this analysis implicated specific clusters of interneurons derived from the caudal and medial ganglionic eminences and medium spiny neurons predominantly localized in the striatum (15). Although interneurons derived from ganglionic eminences were also enriched in SCZ, stronger signals were observed for amygdala excitatory and hippocampal neurons, while medium spiny neurons were not enriched in depression (19). These results suggest disorder-specific enrichment, which has not previously been shown in lesser-powered datasets.

In addition to enrichment in brain tissue, GWAS signals from the latest BD GWAS were also enriched in the enteroendocrine cells of the large intestine and delta cells of the pancreas in murine single-cell datasets. This enrichment remained significant after cross-dataset conditional analyses with a murine brain tissue dataset, which indicates that the enrichment is not the result of overlapping genes between these cell types and those expressed in neuronal cells (15). Interestingly, short-chain fatty acids have been shown to stimulate enteroendocrine cells leading to serotonin production in the colon, which in turn results in increased serotonin in systemic circulation and in the brain and is a proposed mechanism that acts at the interface of the gut-brain axis (20,21). Interestingly, lithium treatment also upregulates short-chain fatty acid–producing bacteria, so this process highlights a potential mechanism of action for the drug (22) and may explain the observed enrichment from GWAS signals. However, these results require validation in human single-cell datasets when these data become available. Moreover, further evaluation of this enrichment based on ascertainment and BD subtype should be investigated given the differences in genetic architecture between these data types as described below.

To better understand the underlying molecular mechanisms of BD, the latest BD GWAS results were subjected to a number of in silico gene mapping strategies to establish a list of credible genes with corroborating evidence (15). The integration of these analyses identified a set of 36 credible genes with evidence for association with BD from at least 3 of the 7 independent approaches (Figure 2B). The *SP4* gene was implicated by 6 of these approaches. This gene is also implicated in SCZ through both common (18) and rare (23) variation, further highlighting the known overlapping genetics of BD and SCZ. Characterization of the temporal expression of these 36 genes showed 2 clusters with distinct expression patterns (Figure 2B). The first cluster showed low prenatal gene expression, with gene expression peaking at birth and remaining stable over the life course. In contrast, the second cluster showed a peak in gene expression during fetal development with a drop in expression before birth. These results are consistent with the neurodevelopmental hypothesis of mental disorders (24).

Although in silico analyses of GWAS loci provide some insight into the underlying biological mechanisms, advances in in vitro models that integrate induced pluripotent stem cells and gene editing methods have the potential to further elucidate the underlying pathobiology. Recent studies utilizing psychiatric cross-disorder (25) and SCZ GWAS cross-disorder (26) data implemented massively parallel variant annotation pipelines for large-scale variant-to-function mapping in relevant cell types. Both studies prioritized hundreds of functional variants and were able to link these to target genes, biological processes, and neuronal physiology (25,26). Importantly, the identified genes only partially overlap with genes identified though in silico fine-mapping strategies, highlighting the benefit of performing such experimental approaches. Implementing a similar approach using the latest well-powered BD GWAS data may provide additional insights into the disorder etiology currently not captured with available in silico tools.

### Genetic Architecture

Using Gaussian mixture models (27), between 8000 and 9000 genetic variants are estimated to influence BD (12). Moreover, using linkage disequilibrium (LD) score regression (28), the single nucleotide polymorphism (SNP)–based heritability (*h*^2^_SNP_) of BD was estimated at approximately 18% on the liability scale, assuming a 2% population prevalence of BD (12). However, when considering the ascertainment source of the cohorts and BD subtypes, the *h*^2^_SNP_ range is relatively large. BD case definitions based on different assessment methods, including (semi)structured clinical interviews (clinical), medical records, registries, and questionnaire data (community) and self-reported surveys (self-reported) were used to define BD ascertainment groups (15). The clinical cohorts contained a significantly greater proportion of BDI cases (83% BDI) when compared with the community cohorts (69% BDI) (15). Furthermore, BD ascertained from clinical cohorts was shown to be more heritable (*h*^2^_SNP_ = 22%) than BD ascertained from community samples (*h*^2^_SNP_ = 5%) or self-report (*h*^2^_SNP_ = 8%) (15). Additionally, BDI was more heritable (*h*^2^_SNP_ = 21%) than BDII (*h*^2^_SNP_ = 11%) (15).

Further investigation by ascertainment and subtype revealed a strong genetic correlation between BD from clinical and community cohorts (*r*_g_ = 0.95) and between BDI and BDII (*r*_g_ = 0.88). The genetic correlation between self-reported BD and BD from community cohorts (*r*_g_ = 0.79) was greater than that observed between self-reported BD and BD from clinical cohorts (*r*_g_ = 0.47). BD from community cohorts was also strongly correlated with both subtypes (BDI *r*_g_ = 0.85, BDII *r*_g_ = 0.95). Similar to BD from clinical cohorts, BDI showed a lower genetic correlation (*r*_g_ = 0.42) with self-reported BD than BDII (*r*_g_ = 0.76); however, covarying for BD subtype negated this difference, suggesting that the differences in genetic architecture are driven by BD subtype (15).

When considering the relationship between BD and other psychiatric disorders, BDII and BD identified in community and self-report cohorts showed stronger correlations with major depressive disorder (MDD), posttraumatic stress disorder (PTSD), attention-deficit/hyperactivity disorder (ADHD), borderline personality disorder, and autism spectrum disorder (ASD). In contrast, BDI and BD from clinical cohorts were more strongly correlated with SCZ (15). While the strong correlation between BDI and SCZ is consistent with previous findings (11,12), these results also demonstrate how the correlation between BD and other psychiatric disorders can vary depending on the inclusion of BDII and broader BD spectrum cases.

These differences in genetic architecture across ascertainment and subtypes were shown to be driven by the proportion of BD subtype within various samples and impacted the replicability of previous BD-associated loci (15). Specifically, previous BD-associated loci that were below the genome-wide significance threshold in the full meta-analysis including self-reported BD were genome-wide significant in the clinical samples and when self-reported BD samples were excluded from the meta-analysis. Similar results related to the inclusion of self-reported case samples in GWAS meta-analyses have been observed in depression, where minimal phenotyping may result in low-specificity association signals and poorer polygenic score (PGS) performance (29–31). Therefore, stratification by BD subtype and careful phenotypic characterization of included samples will be important for BD genetic studies in the future.

### Prediction of BD

With increased discovery of common variant signals, the phenotypic variance explained by the genome-wide BD PGS has also dramatically increased. Previous PGSs explained approximately 4% of the phenotypic variance in BD (11,12), while the latest BD PGS was shown to explain approximately 9% of the phenotypic variance (area under the curve [AUC] = 0.70; 95% CI, 0.67–0.73) (15). Thus, although the BD liability explained remains insufficient for diagnostic prediction in the general population (14), the utility of the PGS has increased sufficiently such that combining it with other predictors of BD risk may improve predictive power, as has been shown for other diseases (32). Nonetheless, more work is needed to define the clinical setting in which PGSs can aid clinical decision making (33,34).

Consistent with the ascertainment- and subtype-related differences in genetic architecture, PGS results were influenced by the inclusion of self-report BD cohorts in the discovery sample (15). Importantly, PGSs derived from the discovery sample excluding the self-report cohorts explained more phenotypic variance in cases of BDI and in clinical cohorts. The inclusion of the self-report cohorts in the discovery sample led to increased variance explained in cases of BDII and in community cohorts. This suggests that the self-report cohorts likely include cases from the wider BD spectrum rather than BDI.

Moreover, evaluation of the BD PGSs in cohorts of East Asian and African ancestry also showed some improvement from previous iterations (12,15), but performance remains poorer than in European ancestry cohorts, as described above. Specifically, the latest BD PGS explains 3% (up from 2%) of the phenotypic variance within Japanese and 2% (approximately the same as the previous iteration PGS) within Korean BD cohorts. In addition, this BD PGS also explains approximately 7% of the phenotypic variance in a Taiwanese BD cohort (15). Furthermore, the BD PGS accounted for approximately 2% of the phenotypic variance (up from 1%) in an African ancestry cohort (15).

**Figure 3.**
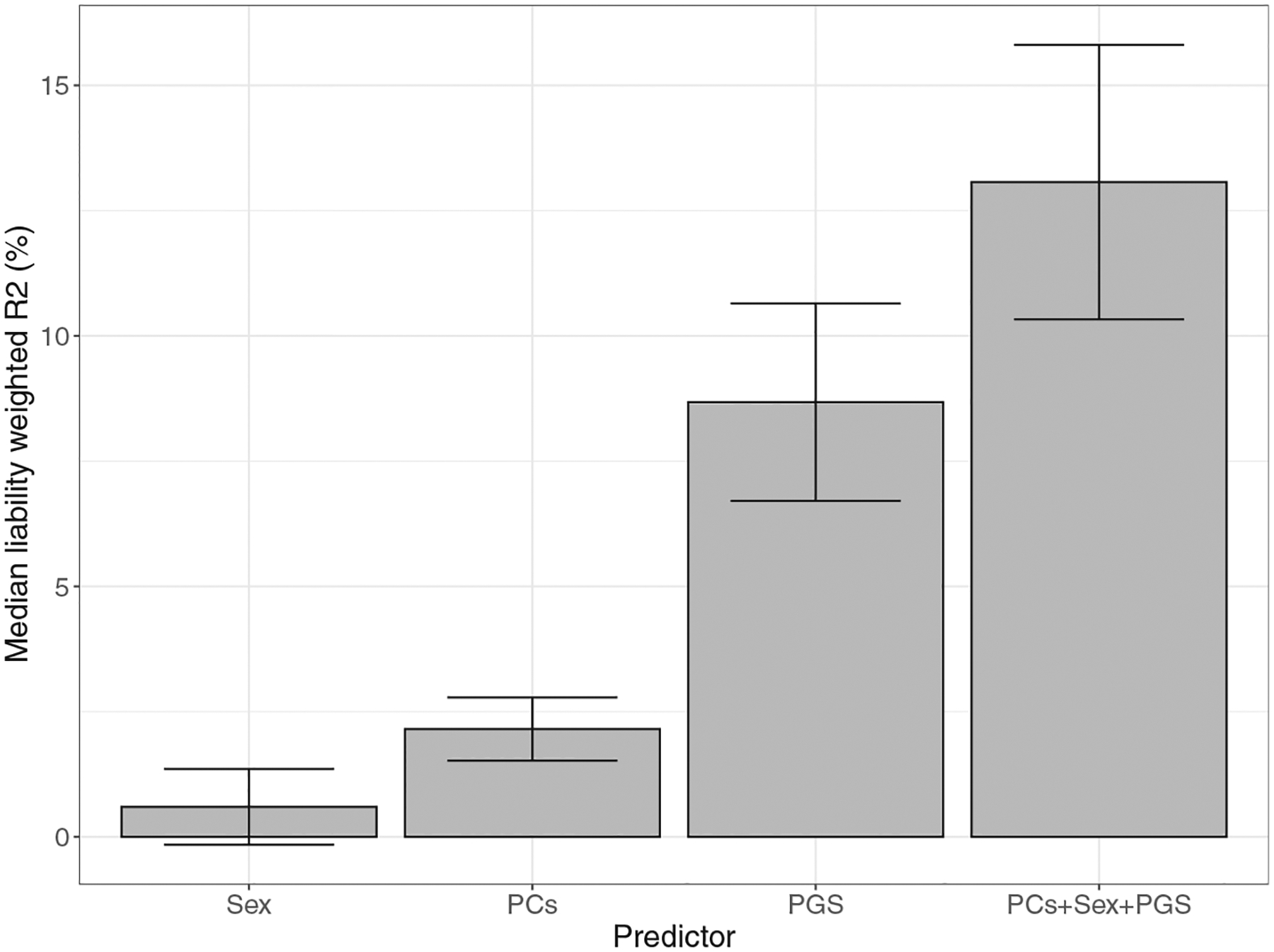
Phenotypic variance in BD in European cohorts explained by sex, the first 5 genetic PCs, and PGS. Variance explained is presented on the liability scale, assuming a 2% population prevalence of BD. Error bars indicate standard errors. All analyses were weighted by the effective *N* per cohort (Table S1). The PGS was derived from the multiancestry meta-analysis excluding self-reported BD (15). BD, bipolar disorder; PC, principal component; PGS, polygenic score.

The models used to estimate the variance explained by the PGSs mentioned above also include the biological sex and the first 5 genetic principal components (PCs) as covariates (15). These covariates are included in the GWAS for discovery of case-control differences to correct for interindividual differences as well as to account for population stratification to avoid spurious findings driven by differences in LD structure between study participants and cohorts. However, for the purpose of parsing effects of specific predictors of the status of BD cases, it would be more pragmatic to further explore the prediction power by including all model variables as predictors instead. Therefore, from the same European samples and models used to establish the variance explained by the PGSs above, we also determined the variance explained by sex, collectively by the first 5 PCs and by the full model, i.e., PGS, sex, and PCs (Supplementary Methods). Using this approach, we show that sex explains approximately 0.6% of the phenotypic variance in BD, while the first 5 PCs explain about 2% (Figure 3 and Table S1), indicative of possible effects of ancestry as well as cohort-related environmental factors. Together, the PGS, sex, and the first 5 PCs explain approximately 13% of the phenotypic variance in BD, with a median AUC of 0.71 (95% CI, 0.68–0.75) (Figure 3 and Table S1).

These new results show that the inclusion of sex and PCs in the prediction model, thereby taking advantage of the variation in LD structure between populations, significantly improves prediction of BD. Furthermore, these results show that even within European populations from different European countries, the variation captured by PCs can be used to improve prediction. This approach may be relevant for future clinical applications of PGSs and should be considered in the development of precision medicine tools in populations of mixed ancestries to avoid health disparities. Today, there is no clinical value of polygenic methods to predict BD, or other mental disorders, at an individual patient level.

### Pharmacogenomics

Current treatment options for BD include pharmacotherapies such as mood stabilizers, antipsychotics, and antidepressants, which are administered in conjunction with psychosocial interventions if possible (1,35). Response to these medications can vary widely between individuals, and some patients may cycle through different medications before they find an effective treatment with minimal side effects. Approximately one-third of patients relapse during the first year of treatment (36). Several lithium response GWASs have been performed (37,38), yielding a few genome-wide significant loci. However, the challenge of assembling sufficiently large and homogeneous samples with standardized measures has, to date, limited the statistical power of treatment response studies. More recently, PGSs derived from an updated meta-analysis were used to assess lithium treatment response in independent cohorts (39). Individuals in the bottom decile of the PGS (lowest genetic liability for lithium nonresponse) were 3.5-fold more likely to respond favorably to lithium than those in the top decile, highlighting the potential for stratification approaches. Moreover, gene-based analyses have identified 36 candidate genes implicating glutamate- and acetylcholine-related biological pathways in lithium response etiology (39). This remains an important area for future research, as understanding treatment response is a critical clinical tool for personalizing and optimizing care in BD. By performing gene-set analysis on known drug targets, GWAS data can be exploited for drug discovery. Applying this analysis to the latest BD GWAS at the drug class set level identified significant enrichment in the targets of antipsychotics and anxiolytics, respectively (15).

## RARE VARIATION

The Bipolar Exome (BipEx) consortium performed whole-exome sequencing in approximately 14,000 BD cases to investigate the burden of ultrarare variants (minor allele count ≤ 5), including damaging missense and protein-truncating variants on BD (40). No exome-wide significant gene associations were identified in the BD dataset alone. However, after combining this dataset with an independent SCZ dataset [Schizophrenia Exome Sequencing Meta-analysis (SCHEMA) consortium (23)], the *AKAP11* gene was identified as a definitive risk gene. To date, none of the loci identified in BD GWASs overlap with the *AKAP11* gene, and it has not been mapped to any loci by in silico gene mapping strategies. Given that the majority of the BipEx sample is diagnosed with BDI (approximately 8000 individuals) and that this dataset was combined with an SCZ dataset, the *AKAP11* association may be more related to the etiology of psychosis rather than to BD. Encouragingly, genes (*n* = 71) annotated to putatively causal fine-mapped SNPs from the latest BD GWAS were enriched for ultrarare variants in BD cases in BipEx (odds ratio [OR] = 1.16; 95% CI, 1.05–1.28; *p* = .002) and in SCZ cases in SCHEMA (OR = 1.21; 95% CI, 1.02–1.43; *p* = .024), highlighting convergence of signal from common and rare variation in BD (15).

## TRANSCRIPTOMICS IN BD

A recent study utilized a multiomics data approach by integrating BD GWAS summary statistics data with single-cell RNA-seq data from embryonic and fetal brain samples, a BD assay for transposase-accessible chromatin using sequencing data in adult brain samples and BD bulk RNA-seq data from adult brain samples (41). They identified a significant association between BD and glial cell types, including astrocytes, oligodendrocyte precursor cells, and microglia. These results confirm previous findings based on the analysis of gene expression, local splicing, transcript isoform expression, and coexpression networks for both protein-coding and noncoding genes in BD, SCZ, and ASD that showed disease-specific alterations in microglial-, astrocyte-, and interferon-response modules (42). Furthermore, a gene module enriched for microglial-associated genes has been shown to be downregulated in BD (43). Despite consistent findings at a transcriptomic level, these studies have often failed to detect significant case-control differential expression of genes implicated by GWASs (42,43). In an attempt to explain this, a recent study analyzed 4 case-control cortical brain RNA-seq datasets (42–44), with a focus on BD GWAS–associated genes (12), to examine patterns of absolute differential gene expression (45). This study identified consistent patterns of differential expression across datasets and observed modules of coexpression between BD GWAS–associated genes. These results suggest that a stoichiometric constraint may apply to many of the BD GWAS–associated genes, whereby the ratios in expression between subsets of genes are kept relatively consistent to ensure healthy brain function. Thus, the underlying molecular pathology of BD may be a result of stoichiometric imbalance rather than abnormal absolute gene expression levels (45). This is consistent with polygenic alterations of brain function in psychiatric disorders (46) and could result in functional abnormalities, such as dysregulated neuronal excitability (47), which may underlie BD (48).

## CLINICAL HETEROGENEITY OF BD

As described above, although the BD subtypes are largely genetically similar, there are distinct differences in their genetic architecture that impacts the results of genetic studies of BD. These differences in genetic architecture and genetic overlap with other traits may help to explain observed differences in comorbidities between the subtypes. Patients with BDII typically present with higher rates of depressive episodes, a history of rapid cycling, and more frequent suicide attempts than patients with BDI (49–52). They also have higher rates of comorbid medical conditions (49,51) and other psychiatric diagnoses (49,52). Despite this, patients with BDII have fewer hospitalizations and increased employment than individuals with a BDI diagnosis (49,51,52).

PGSs derived from GWASs of related diseases and traits have shown some utility in dissecting this clinical heterogeneity of BD. For example, greater genetic liability for SCZ is associated with BDI (53) and with psychosis in patients with BD (54), while greater liability for depression, PTSD, and ADHD are associated with rapid cycling (54). These PGS results are consistent with the differences in genetic architecture that have been observed between the BD subtypes (14). Moreover, recent findings have demonstrated that a BD-PRS is positively correlated with interepisode remission (55) and functional outcomes (56) but negatively correlated with comorbid anxiety disorders, while an SCZ-PRS is positively associated with psychotic symptoms during mood episodes. This further highlights the role of genetic loadings in shaping BD subphenotypes.

Given the clinical differences in disease course between the subtypes, it is unsurprising that treatment regimens are also different. Specifically, patients with BDII are more often prescribed antidepressants but receive less treatment with lithium or antipsychotics (49–52).

Despite seemingly well-defined and characterized clinical heterogeneity between the BD subtypes, systematic differences in the evaluation and diagnosis of the subtypes can also impact genetic studies. A recent study investigated the genetic correlations between BD subtypes and SCZ and MDD in East Asian cohorts (57). They observed a lower-than-expected genetic correlation between BDI and SCZ (*r*_g_ = 0.36), while the genetic correlation between BDII and MDD (*r*_g_ = 0.68) was consistent with previous findings in European datasets. The authors postulated that a lower proportion of BDI cases with psychosis, due to different diagnostic trends within East Asian clinicians, is the likely reason for this discordance. A similar finding of systematic heterogeneity at the clinical level influencing genetic results was also observed when considering age at onset of BD, where both assessment strategy and geographic location were shown to impact heritability estimates (58). Furthermore, the polygenic architecture of clinically ascertained cohorts may change over time, potentially reflecting secular trends and differing diagnostic traditions. For example, one study found significant associations between the year of diagnosis and PGSs for BD, MDD, and ADHD, with the most pronounced trends observed for BDII. By contrast, BDI remained relatively stable across the period from 1972 to 2016 (59).

## CONCLUSIONS

Combining the full BD spectrum, and thereby increasing sample size and power, increases genetic discovery and improves the ability to identify the biological processes that underlie the general characteristics of the disorder. Extensive progress has been made in identifying common variant signals for BD, and the PGS from the latest GWAS may provide some clinical utility if combined with other risk factors for BD. Furthermore, the most recent GWAS of BD has identified 36 credible BD genes, and functional analyses implicated specific enrichment in GABAergic interneurons and medium spiny neurons. In addition, enrichment in enteroendocrine cells of the large intestine and delta cells of the pancreas increased our understanding of the neurobiology of BD

However, there are also clear indications that the BD subtypes differ in their genetic architecture. Therefore, genetic studies across the subtypes of the BD spectrum will identify subtype-specific signals and reveal subtype-specific biological mechanisms. Notably, subtype-specific GWAS sample sizes have not increased at the same rate as BD cases, because diagnoses may be determined from medical records, registries, questionnaires, and self-report that do not often include this level of detail. More concerted efforts are required to obtain BD subtype information for participants included in GWASs to increase power for subtype-specific analyses. In addition, careful assessment of social, cultural, geographic, and other systematic differences that may impact patient assessment will be necessary to ensure accurate inclusion of diverse ancestral groups and global representation in genetic studies of BD in the future.

## Supplementary Material

1

2

Supplementary material cited in this article is available online at https://doi.org/10.1016/j.biopsych.2025.05.020.
