# Supplementary material for: New Genomics Discoveries Across the Bipolar Disorder Spectrum Implicate Neurobiological and Developmental Pathways
Source: Biol Psychiatry. Author manuscript; Available in PMC 2026 May 27. (PMC13213455; doi:10.1016/j.biopsych.2025.05.020)
Supplement: 1 [file NIHMS2177177-supplement-1.pdf]

SUPPLEMENTARY INFORMATION

New Genomics Discoveries Across the Bipolar Disorder Spectrum Implicate Neurobiological and Developmental Pathways

O'Connell *et al.*

Supplementary Methods

We used PRS-CS-auto (1) to compute polygenic risk scores in target cohorts, using a discovery GWAS where the target cohort was left out. We used the EUR LD reference panel based on UK BioBank data as provided by PRS-CS developers (https://github.com/getian107/PRScs). Raw scores were standardised to Z scores, and with sex and the first five PCs and any others as required were included in the logistic regression model, via the glm() function in R (2), with family=binomial and link=logit. The variance explained by each predictor (R^2) was first converted to Nagelkerke's pseudo-R^2 via the fmsb package in R (https://cran.r-project.org/web/packages/fmsb/index.html), then converted to the liability scale to account for proportion of cases in each cohort and the population prevalence of BD (3). We provide R^2 values for BD assuming a population prevalence of 2%, based upon a recent multinational survey (4). The weighted average R^2 values were then calculated using the effective sample size for each cohort. The area under the curve (AUC) statistic was calculated via the pROC package in R (https://cran.r-project.org/web/packages/pROC/index.html), for which we performed a training and testing procedure by taking 80% of the individuals in a given cohort on which to train the model, and tested the predictability in the remaining 20% of individuals. Ten random samplings of training and testing sets were performed in all cohorts, and the median AUC after all permutations is provided in the Supplementary Table. The median confidence intervals for the AUC were similarly averaged across the ten random permutations.

Methods References

1. Ge T, Chen CY, Ni Y, Feng YA, Smoller JW (2019): Polygenic prediction via Bayesian regression and continuous shrinkage priors. *Nature communications*. 10:1776.
2. R Core Team (2020): R: A language and environment for statistical computing. *R Foundation for Statistical Computing, Vienna, Austria*.
3. Lee SH, Goddard ME, Wray NR, Visscher PM (2012): A better coefficient of determination for genetic profile analysis. *Genetic epidemiology*. 36:214-224.
4. McGrath JJ, Al-Hamzawi A, Alonso J, Altwaijri Y, Andrade LH, Bromet EJ, et al. (2023): Age of onset and cumulative risk of mental disorders: a cross-national analysis of population surveys from 29 countries. *Lancet Psychiatry*. 10:668-681.

Bipolar Disorder Working Group of the Psychiatric Genomics Consortium Names and Affiliations

Kevin S. O'Connell[1,2], Maria Koromina[3,4,5] , Tracey van der Veen[6] , Toni Boltz[7], Friederike S. David[8,9], Jessica Mei Kay Yang[10], Keng-Han Lin[11], Xin Wang[11], Jonathan R. I. Coleman[12,13], Brittany L. Mitchell[14,15], Caroline C. McGrouther[16], Aaditya V. Rangan[16,17], Penelope A. Lind[14,15,18], Elise Koch[1,2], Arvid Harder[19], Nadine Parker[1,2], Jaroslav Bendl[3,5,20,21], Kristina Adorjan[22,23,24], Esben Agerbo[25,26,27], Diego Albani[28], Silvia Alemany[29,30,31], Ney Alliey-Rodriguez[32,33], Thomas D. Als[25,34,35], Till F. M. Andlauer[36], Anastasia Antoniou[37], Helga Ask[38,39], Nicholas Bass[6], Michael Bauer[40], Eva C. Beins[8], Tim B. Bigdeli[41,42,43,44], Carsten Bøcker Pedersen[25,26,27], Marco P. Boks[45], Sigrid Børte[46,47,48], Rosa Bosch[29,49], Murielle Brum[50], Ben M. Brumpton[51], Nathalie Brunkhorst-Kanaan[50], Monika Budde[22], Jonas Bybjerg-Grauholm[25,52], William Byerley[53], Judit Cabana-Domínguez[29,30,31], Murray J. Cairns[54,55], Bernardo Carpiniello[56], Miquel Casas[49,57,58], Pablo Cervantes[59], Chris Chatzinakos[41,43], Hsi-Chung Chen[60,61], Tereza Clarence[3,5,20,21], Toni-Kim Clarke[62], Isabelle Claus[8], Brandon Coombes[63], Elizabeth C. Corfield[38,64,65], Cristiana Cruceanu[59,66], Alfredo Cuellar-Barboza[67,68], Piotr M. Czerski[69], Konstantinos Dafnas[37], Anders M. Dale[70], Nina Dalkner[71], Franziska Degenhardt[8,72], J. Raymond DePaulo[73], Srdjan Djurovic[74,75], Ole Kristian Drange[2,76], Valentina Escott-Price[10], Ayman H. Fanous[77,78,79], Frederike T. Fellendorf[71], I. Nicol Ferrier[80], Liz Forty[10], Josef Frank[81], Oleksandr Frei[1,47], Nelson B. Freimer[82,83], John F. Fullard[3,5,20,21], Julie Garnham[84], Ian R. Gizer[85], Scott D. Gordon[86], Katherine Gordon-Smith[87], Tiffany A. Greenwood[88], Jakob Grove[25,89,90,91], José Guzman-Parra[92], Tae Hyon Ha[93,94], Tim Hahn[95], Magnus Haraldsson[96,97], Martin Hautzinger[98], Alexandra Havdahl[38,39,64], Urs Heilbronner[22], Dennis Hellgren[19], Stefan Herms[8,99,100], Ian B. Hickie[101], Per Hoffmann[8,99,100], Peter A. Holmans[10], Ming-Chyi Huang[102], Masashi Ikeda[103,104], Stéphane Jamain[105], Jessica S. Johnson[3,5,106], Lina Jonsson[107], Janos L. Kalman[22,23], Yoichiro Kamatani[108,109], James L. Kennedy[110,111,112,113], Euitae Kim[93,94,114], Jaeyoung Kim[93,115], Sarah Kittel-Schneider[116,117], James A. Knowles[118], Manolis Kogevinas[119], Thorsten M. Kranz[50], Kristi Krebs[120], Steven A. Kushner[121], Catharina Lavebratt[122,123], Jacob Lawrence[124], Markus Leber[125], Heon-Jeong Lee[126], Calwing Liao[127,128], Susanne Lucae[129], Martin Lundberg[122,123], Donald J. MacIntyre[130], Wolfgang Maier[131], Adam X. Maihofer[88,132], Dolores Malaspina[3,5], Mirko Manchia[56,133], Eirini Maratou[134], Lina Martinsson[135,136], Manuel Mattheisen[25,34,35,117,137], Nathaniel W. McGregor[138], Melvin G. McInnis[139], James D. McKay[140], Helena Medeiros[141], Andreas Meyer-Lindenberg[142,143], Vincent Millischer[122,123,144,145], Derek W. Morris[146], Paraskevi Moutsatsou[134], Thomas W. Mühleisen[99,147], Claire O'Donovan[84], Catherine M. Olsen[148], Georgia Panagiotaropoulou[149], Sergi Papiol[22,23,29], Antonio F. Pardiñas[10], Hye Youn Park[93,94], Amy Perry[87], Andrea Pfennig[40], Claudia Pisanu[150], James B. Potash[73], Digby Quested[151,152], Mark H. Rapaport[153], Eline J. Regeer[154], John P. Rice[155], Margarita Rivera[156,157,158], Eva C. Schulte[8,22,131], Fanny Senner[22,23], Alexey Shadrin[1,2,159], Paul D. Shilling[88], Engilbert Sigurdsson[96,97], Lisa Sindermann[8], Lea Sirignano[81], Dan Siskind[160], Claire Slaney[84], Laura G. Sloofman[3,5], Olav B. Smeland[1,2], Daniel J. Smith[161], Janet L. Sobell[162], Maria Soler Artigas[29,30,31,163], Dan J. Stein[164], Frederike Stein[9], Mei-Hsin Su[165], Heejong Sung[166], Beata Świątkowska[167], Chikashi Terao[109], Markos Tesfaye[1,2,75], Martin Tesli[1,2,168], Thorgeir E. Thorgeirsson[169], Jackson G. Thorp[14], Claudio Toma[170,171,172], Leonardo Tondo[173], Paul A. Tooney[174], Shih-Jen Tsai[175,176], Evangelia Eirini Tsermpini[84], Marquis P. Vawter[177], Helmut

Vedder[178], Annabel Vreeker[45,179,180], James T. R. Walters[10], Bendik S. Winsvold[48,181,182], Stephanie H. Witt[81], Hong-Hee Won[115,183], Robert Ye[127,128], Allan H. Young[184,185], Peter P. Zandi[73], Lea Zillich[81], 23andMe Research Team[186], Estonian Biobank research team[186], Genoplan Research Team[186], HUNT All-In Psychiatry[186], PGC-FG Single cell working group[186], Genomic Psychiatry Cohort (GPC) Investigators[186], Cooperative Studies Program (CSP) #572[186], Million Veteran Program (MVP)[186], Rolf Adolfsson[187], Martin Alda[84,188], Lars Alfredsson[189], Lena Backlund[122,123], Bernhard T. Baune[190,191,192], Frank Bellivier[193,194], Susanne Bengesser[71], Wade H. Berrettini[195], Joanna M. Biernacka[63,68], Michael Boehnke[196], Anders D. Børglum[25,89,90], Gerome Breen[12,13], Vaughan J. Carr[171], Stanley Catts[197], Sven Cichon[8,99,100,147], Aiden Corvin[198], Nicholas Craddock[10], Udo Dannlowski[95], Dimitris Dikeos[199], Bruno Etain[193,194], Panagiotis Ferentinos[12,37], Mark Frye[68], Janice M. Fullerton[170,200], Micha Gawlik[117], Elliot S. Gershon[32,201], Fernando S. Goes[73], Melissa J. Green[170,171], Maria Grigoroiu-Serbanescu[202], Joanna Hauser[203], Frans A. Henskens[204], Jens Hjerling-Leffler[205], David M. Hougaard[25,52], Kristian Hveem[51,206], Nakao Iwata[104], Ian Jones[10], Lisa A. Jones[87], René S. Kahn[3,45], John R. Kelsoe[88], Tilo Kircher[9], George Kirov[10], Po-Hsiu Kuo[60,207], Mikael Landén[19,107], Marion Leboyer[105], Qingqin S. Li[208,209], Jolanta Lissowska[210], Christine Lochner[211], Carmel Loughland[212], Jurjen J. Luykx[213,214], Nicholas G. Martin[86,215], Carol A. Mathews[216], Fermin Mayoral[92], Susan L. McElroy[217], Andrew M. McIntosh[130], Francis J. McMahon[166], Sarah E. Medland[14,218,219], Ingrid Melle[1,220], Lili Milani[120], Philip B. Mitchell[171], Gunnar Morken[221,222], Ole Mors[25,223], Preben Bo Mortensen[25,224], Bertram Müller-Myhsok[129,225,226], Richard M. Myers[227], Woojae Myung[93,94], Benjamin M. Neale[127,128,228], Caroline M. Nievergelt[88,132], Merete Nordentoft[25,229], Markus M. Nöthen[8], John I. Nurnberger[230], Michael C. O'Donovan[10], Ketil J. Oedegaard[231,232], Tomas Olsson[233], Michael J. Owen[10], Sara A. Paciga[234], Christos Pantelis[192,235,236], Carlos N. Pato[237], Michele T. Pato[237], George P. Patrinos[238,239,240,241], Joanna M. Pawlak[203], Josep Antoni Ramos-Quiroga[29,30,31,57], Andreas Reif[50], Eva Z. Reininghaus[71], Marta Ribasés[29,30,31,163], Marcella Rietschel[81], Stephan Ripke[127,128,149], Guy A. Rouleau[242,243], Panos Roussos[3,5,20,21,244], Takeo Saito[104], Ulrich Schall[245,246], Martin Schalling[122,123], Peter R. Schofield[170,200], Thomas G. Schulze[22,73,81,247,248], Laura J. Scott[196], Rodney J. Scott[249,250], Alessandro Serretti[251,252,253], Jordan W. Smoller[128,254,255], Alessio Squassina[150], Eli A. Stahl[3,5,228], Hreinn Stefansson[169], Kari Stefansson[169,256], Eystein Stordal[257,258], Fabian Streit[81,142,259], Patrick F. Sullivan[19,260,261], Gustavo Turecki[262], Arne E. Vaaler[263], Eduard Vieta[264], John B. Vincent[110], Irwin D. Waldman[265], Cynthia S. Weickert[170,171,266], Thomas W. Weickert[170,171,266], Thomas Werge[25,267,268,269], David C. Whiteman[148], John-Anker Zwart[47,48,181], Howard J. Edenberg[270,271], Andrew McQuillin[6], Andreas J. Forstner[8,147], Niamh Mullins[3,4,5], Arianna Di Florio[10,261], Roel A. Ophoff[7,82,83], Ole A. Andreassen[1,2]

Affiliations

[1]Division of Mental Health and Addiction, Oslo University Hospital, Oslo, Norway. [2]NORMENT, University of Oslo, Oslo, Norway. [3]Department of Psychiatry, Icahn School of Medicine at Mount Sinai, New York, NY, USA. [4]Charles Bronfman Institute for Personalized Medicine, Icahn School of Medicine at Mount Sinai, New York, NY, USA. [5]Department of Genetics and Genomic Sciences, Icahn School of Medicine at Mount Sinai, New York, NY, USA. [6]Division of Psychiatry, University College London, London, UK. [7]Department of Human Genetics, David Geffen School of Medicine, University of California Los Angeles, Los Angeles, CA, USA. [8]Institute of Human Genetics, University of Bonn, School of Medicine

and University Hospital Bonn, Bonn, Germany. [9]Department of Psychiatry and Psychotherapy, University of Marburg, Marburg, Germany. [10]Centre for Neuropsychiatric Genetics and Genomics, Division of Psychological Medicine and Clinical Neurosciences, Cardiff University, Cardiff, UK. [11]23andMe, Inc., Sunnyvale, CA, USA. [12]Social, Genetic and Developmental Psychiatry Centre, King's College London, London, UK. [13]NIHR Maudsley BRC, King's College London, London, UK. [14]Mental Health and Neuroscience, QIMR Berghofer Medical Research Institute, Brisbane, QLD, Australia. [15]School of Biomedical Sciences and Faculty of Medicine, The University of Queensland, Brisbane, QLD, Australia. [16]New York University, New York, NY, USA. [17]Flatiron Institute, New York, NY, USA. [18]School of Biomedical Sciences, Queensland University of Technology, Brisbane, QLD, Australia. [19]Department of Medical Epidemiology and Biostatistics, Karolinska Institutet, Stockholm, Sweden. [20]Center for Disease Neurogenomics, Icahn School of Medicine at Mount Sinai, New York, NY, USA. [21]Friedman Brain Institute, Icahn School of Medicine at Mount Sinai, New York, NY, USA. [22]Institute of Psychiatric Phenomics and Genomics (IPPG), LMU University Hospital, LMU Munich, Munich, Germany. [23]Department of Psychiatry and Psychotherapy, University Hospital, LMU Munich, Munich, Germany. [24]University Hospital of Psychiatry and Psychotherapy, University of Bern, Switzerland. [25]iPSYCH, The Lundbeck Foundation Initiative for Integrative Psychiatric Research, Denmark. [26]National Centre for Register-Based Research, Aarhus University, Aarhus, Denmark. [27]Centre for Integrated Register-based Research, Aarhus University, Aarhus, Denmark. [28]Department of Neuroscience, Istituto Di Ricerche Farmacologiche Mario Negri IRCCS, Milano, Italy. [29]Instituto de Salud Carlos III, Biomedical Network Research Centre on Mental Health (CIBERSAM), Madrid, Spain. [30]Department of Psychiatry, Hospital Universitari Vall d´Hebron, Barcelona, Spain. [31]Psychiatric Genetics Unit, Group of Psychiatry Mental Health and Addictions, Vall d´Hebron Research Institut (VHIR), Universitat Autònoma de Barcelona, Barcelona, Spain. [32]Department of Psychiatry and Behavioral Neuroscience, University of Chicago, Chicago, IL, USA. [33]Northwestern University, Chicago, IL, USA. [34]iSEQ, Center for Integrative Sequencing, Aarhus University, Aarhus, Denmark. [35]Department of Biomedicine - Human Genetics, Aarhus University, Aarhus, Denmark. [36]Department of Neurology, Klinikum rechts der Isar, School of Medicine, Technical University of Munich, Munich, Germany. [37]National and Kapodistrian University of Athens, 2nd Department of Psychiatry, Attikon General Hospital, Athens, Greece. [38]PsychGen Centre for Genetic Epidemiology and Mental Health, Norwegian Institute of Public Health, Oslo, Norway. [39]PROMENTA Research Centre, Department of Psychology, University of Oslo, Norway. [40]Department of Psychiatry and Psychotherapy, University Hospital Carl Gustav Carus, Technische Universität Dresden, Dresden, Germany. [41]Department of Psychiatry and Behavioral Sciences, SUNY Downstate Health Sciences University, Brooklyn, NY, USA. [42]VA NY Harbor Healthcare System, Brooklyn, NY, USA. [43]Institute for Genomics in Health, SUNY Downstate Health Sciences University, Brooklyn, NY, USA. [44]Department of Epidemiology and Biostatistics, School of Public Health, SUNY Downstate Health Sciences University, Brooklyn, NY, USA. [45]Psychiatry, Brain Center UMC Utrecht, Utrecht, The Netherlands. [46]Research and Communication Unit for Musculoskeletal Health, Division of Clinical Neuroscience, Oslo University Hospital, Ullevål, Oslo, Norway. [47]Institute of Clinical Medicine, University of Oslo, Oslo, Norway. [48]HUNT Center for Molecular and Clinical Epidemiology, Department of Public Health and Nursing, Faculty of Medicine and Health Sciences, Norwegian University of Science and Technology, Trondheim, Norway. [49]Programa SJD MIND Escoles, Hospital Sant Joan de Déu, Institut de Recerca Sant Joan de Déu, Esplugues de Llobregat, Spain. [50]Department of Psychiatry, Psychosomatic

Medicine and Psychotherapy, University Hospital Frankfurt, Frankfurt am Main, Germany. [51]K. G. Jebsen Center for Genetic Epidemiology, Department of Public Health and Nursing, Faculty of Medicine and Health Sciences, Norwegian University of Science and Technology, Trondheim, Norway. [52]Center for Neonatal Screening, Department for Congenital Disorders, Statens Serum Institut, Copenhagen, Denmark. [53]Psychiatry, University of California San Francisco, San Francisco, CA, USA. [54]School of Biomedical Sciences and Pharmacy, The University of Newcastle, Callaghan, NSW, Australia. [55]Precision Medicine Research Program, Hunter Medical Research Institute, New Lambton, NSW, Australia. [56]Section of Psychiatry, Department of Medical Sciences and Public Health, University of Cagliari, Italy. [57]Department of Psychiatry and Forensic Medicine, Universitat Autònoma de Barcelona, Barcelona, Spain. [58]Fundació Privada d'Investigació Sant Pau (FISP), Barcelona, Spain. [59]Department of Psychiatry, Mood Disorders Program, McGill University Health Center, Montreal, QC, Canada. [60]Department of Psychiatry, National Taiwan University Hospital, Taipei, Taiwan. [61]Department of Psychiatry, College of Medicine, National Taiwan University, Taipei, Taiwan. [62]Division of Psychiatry, University of Edinburgh, Edinburgh, UK. [63]Department of Quantitative Health Sciences Research, Mayo Clinic, Rochester, MN, USA. [64]Nic Waals Institute, Lovisenberg Diaconal Hospital, Oslo, Norway. [65]Department of Genetics and Bioinformatics, Norwegian Institute of Public Health, Oslo, Norway. [66]Department of Physiology and Pharmacology, Karolinska Institutet, Stockholm, Sweden. [67]Department of Psychiatry, Universidad Autonoma de Nuevo Leon, Monterrey, Mexico. [68]Department of Psychiatry and Psychology, Mayo Clinic, Rochester, MN, USA. [69]Department of Psychiatry, Laboratory of Psychiatric Genetics, Poznan University of Medical Sciences, Poznan, Poland. [70]Center for Multimodal Imaging and Genetics, Departments of Neurosciences, Radiology, and Psychiatry, University of California, San Diego, CA, USA. [71]Medical University of Graz, Division of Psychiatry and Psychotherapeutic Medicine, Graz, Austria. [72]Department of Child and Adolescent Psychiatry, Psychosomatics and Psychotherapy, University Hospital Essen, University of Duisburg-Essen, Duisburg, Germany. [73]Department of Psychiatry and Behavioral Sciences, Johns Hopkins University School of Medicine, Baltimore, MD, USA. [74]Department of Medical Genetics, Oslo University Hospital Ullevål, Oslo, Norway. [75]Department of Clinical Science, University of Bergen, Bergen, Norway. [76]Department of Psychiatry, Sørlandet Hospital, Kristiansand/Arendal, Norway. [77]Department of Psychiatry, University of Arizona College of Medicine-Phoenix, Phoenix, AZ, USA. [78]Carl T. Hayden Veterans Affairs Medical Center, Phoenix, AZ, USA. [79]Banner-University Medical Center, Phoenix, AZ, USA. [80]Academic Psychiatry, Newcastle University, Newcastle upon Tyne, UK. [81]Department of Genetic Epidemiology in Psychiatry, Central Institute of Mental Health, Medical Faculty Mannheim, Heidelberg University, Mannheim, Germany. [82]Center for Neurobehavioral Genetics, Semel Institute for Neuroscience and Human Behavior, Los Angeles, CA, USA. [83]Department of Psychiatry and Biobehavioral Science, Semel Institute, David Geffen School of Medicine, University of California, Los Angeles, Los Angeles, CA, USA. [84]Department of Psychiatry, Dalhousie University, Halifax, NS, Canada. [85]Department of Psychological Sciences, University of Missouri, Columbia, MO, USA. [86]Genetics and Computational Biology, QIMR Berghofer Medical Research Institute, Brisbane, QLD, Australia. [87]Psychological Medicine, University of Worcester, Worcester, UK. [88]Department of Psychiatry, University of California San Diego, La Jolla, CA, USA. [89]Department of Biomedicine and the iSEQ Center, Aarhus University, Aarhus, Denmark. [90]Center for Genomics and Personalized Medicine, CGPM, Aarhus, Denmark. [91]Bioinformatics Research Centre, Aarhus University, Aarhus, Denmark. [92]Mental Health Department, University Regional Hospital, Biomedicine Institute (IBIMA), Málaga,

Spain. [93]Department of Neuropsychiatry, Seoul National University Bundang Hospital, Seongnam, Republic of Korea. [94]Department of Neuropsychiatry, Seoul National University College of Medicine, Seoul, Republic of Korea. [95]Institute for Translational Psychiatry, University of Münster, Münster, Germany. [96]Faculty of Medicine, Department of Psychiatry, School of Health Sciences, University of Iceland, Reykjavik, Iceland. [97]Landspitali University Hospital, Reykjavik, Iceland. [98]Department of Psychology, Eberhard Karls Universität Tübingen, Tubingen, Germany. [99]Department of Biomedicine, University of Basel, Basel, Switzerland. [100]Institute of Medical Genetics and Pathology, University Hospital Basel, Basel, Switzerland. [101]Brain and Mind Centre, The University of Sydney, Sydney, NSW, Australia. [102]Department of Psychiatry, Taipei City Psychiatric Center, Taipei City Hospital, Taipei, Taiwan. [103]Department of Psychiatry, Nagoya University Graduate School of Medicine, Nagoya, Japan. [104]Department of Psychiatry, Fujita Health University School of Medicine, Toyoake, Japan. [105]Univ Paris Est Créteil, INSERM, IMRB, Translational Neuropsychiatry, Créteil, France. [106]Department of Psychiatry, UNC Chapel Hill School of Medicine, University of North Carolina at Chapel Hill, Chapel Hill, NC, USA. [107]Institute of Neuroscience and Physiology, University of Gothenburg, Gothenburg, Sweden. [108]Laboratory of Complex Trait Genomics, Department of Computational Biology and Medical Sciences, Graduate School of Frontier Sciences, The University of Tokyo, Tokyo, Japan. [109]Laboratory for Statistical and Translational Genetics, RIKEN Center for Integrative Medical Sciences, Yokohama, Japan. [110]Campbell Family Mental Health Research Institute, Centre for Addiction and Mental Health, Toronto, ON, Canada. [111]Neurogenetics Section, Centre for Addiction and Mental Health, Toronto, ON, Canada. [112]Department of Psychiatry, University of Toronto, Toronto, ON, Canada. [113]Institute of Medical Sciences, University of Toronto, Toronto, ON, Canada. [114]Department of Brain and Cognitive Sciences, Seoul National University College of Natural Sciences, Seoul, Republic of Korea. [115]Samsung Advanced Institute for Health Sciences and Technology (SAIHST), Sungkyunkwan University, Samsung Medical Center, Seoul, Republic of Korea. [116]Department of Psychiatry and Neurobehavioral Science, University College Cork, Cork, Ireland. [117]Department of Psychiatry, Psychosomatics and Psychotherapy, Center of Mental Health, University Hospital Würzburg, Würzburg, Germany. [118]Human Genetics Institute of New Jersey, Rutgers University, Piscataway, NJ, USA. [119]ISGlobal, Barcelona, Spain. [120]Estonian Genome Centre, Institute of Genomics, University of Tartu, Tartu, Estonia. [121]Department of Psychiatry, Erasmus MC, University Medical Center Rotterdam, Rotterdam, The Netherlands. [122]Translational Psychiatry, Department of Molecular Medicine and Surgery, Karolinska Institutet, Stockholm, Sweden. [123]Center for Molecular Medicine, Karolinska University Hospital, Stockholm, Sweden. [124]Psychiatry, North East London NHS Foundation Trust, Ilford, UK. [125]Clinic for Psychiatry and Psychotherapy, University Hospital Cologne, Cologne, Germany. [126]Department of Psychiatry, Korea University College of Medicine, Seoul, Republic of Korea. [127]Analytic and Translational Genetics Unit, Massachusetts General Hospital, Boston, MA, USA. [128]Stanley Center for Psychiatric Research, Broad Institute, Cambridge, MA, USA. [129]Department of Translational Research in Psychiatry, Max Planck Institute of Psychiatry, Munich, Germany. [130]Division of Psychiatry, Centre for Clinical Brain Sciences, The University of Edinburgh, Edinburgh, UK. [131]Department of Psychiatry and Psychotherapy, University of Bonn, School of Medicine and University Hospital Bonn, Bonn, Germany. [132]Research/Psychiatry, Veterans Affairs San Diego Healthcare System, San Diego, CA, USA. [133]Unit of Clinical Psychiatry, University Hospital Agency of Cagliari, Cagliari, Italy. [134]National and Kapodistrian University of Athens, Medical School, Clinical Biochemistry Laboratory, Attikon General Hospital, Athens, Greece. [135]Department of Clinical Neuroscience, Karolinska Institutet, Stockholm, Sweden. [136]Centre

for Psychiatry Research, SLSO Region Stockholm, Sweden. [137]Department of Clinical Neuroscience, Centre for Psychiatry Research, Karolinska Institutet, Stockholm, Sweden. [138]Human and Systems Genetics Working Group, Department of Genetics, Stellenbosch University, Stellenbosch, South Africa. [139]Department of Psychiatry, University of Michigan, Ann Arbor, MI, USA. [140]Genetic Cancer Susceptibility Group, International Agency for Research on Cancer, Lyon, France. [141]Institute for Genomic Health, SUNY Downstate Medical Center College of Medicine, Brooklyn, NY, USA. [142]Department of Psychiatry and Psychotherapy, Central Institute of Mental Health, Medical Faculty Mannheim, University of Heidelberg, Mannheim, Germany. [143]German Centre for Mental Health (DZPG), Germany. [144]Department of Psychiatry and Psychotherapy, Clinical Division of General Psychiatry, Medical University of Vienna, Austria. [145]Comprehensive Center for Clinical Neurosciences and Mental Health, Medical University of Vienna, Vienna, Austria. [146]Centre for Neuroimaging and Cognitive Genomics (NICOG), School of Biological and Chemical Sciences, University of Galway, Galway, Ireland. [147]Institute of Neuroscience and Medicine (INM-1), Research Centre Jülich, Jülich, Germany. [148]Population Health, QIMR Berghofer Medical Research Institute, Brisbane, QLD, Australia. [149]Department of Psychiatry and Psychotherapy, Charité - Universitätsmedizin, Berlin, Germany. [150]Department of Biomedical Sciences, University of Cagliari, Italy. [151]Oxford Health NHS Foundation Trust, Warneford Hospital, Oxford, UK. [152]Department of Psychiatry, University of Oxford, Warneford Hospital, Oxford, UK. [153]Department of Psychiatry and Behavioral Sciences, Emory University School of Medicine, Atlanta, GA, USA. [154]Outpatient Clinic for Bipolar Disorder, Altrecht, Utrecht, The Netherlands. [155]Department of Psychiatry, Washington University in Saint Louis, Saint Louis, MO, USA. [156]Department of Biochemistry and Molecular Biology II, Faculty of Pharmacy, University of Granada, Granada, Spain. [157]Institute of Neurosciences ´Federico Olóriz´, Biomedical Research Center (CIBM), University of Granada, Granada, Spain. [158]Instituto de Investigación Biosanitaria ibs.GRANADA, Granada, Spain. [159]KG Jebsen Centre for Neurodevelopmental disorders, University of Oslo, Oslo, Norway. [160]Faculty of Medicine, University of Queensland, Brisbane, QLD, Australia. [161]Division of Psychiatry, Centre for Clinical Brain Sciences, University of Edinburgh, Edinburgh, UK. [162]Psychiatry and the Behavioral Sciences, University of Southern California, Los Angeles, CA, USA. [163]Department of Genetics, Microbiology, and Statistics, Faculty of Biology, Universitat de Barcelona, Barcelona, Spain. [164]SAMRC Unit on Risk and Resilience in Mental Disorders, Dept of Psychiatry and Neuroscience Institute, University of Cape Town, Cape Town, South Africa. [165]Virginia Institute for Psychiatric and Behavioral Genetics, Virginia Commonwealth University, Richmond, VA, USA. [166]Human Genetics Branch, Intramural Research Program, National Institute of Mental Health, Bethesda, MD, USA. [167]Department of Environmental Epidemiology, Nofer Institute of Occupational Medicine, Lodz, Poland. [168]Department of Mental Disorders, Norwegian Institute of Public Health, Oslo, Norway. [169]deCODE Genetics / Amgen, Reykjavik, Iceland. [170]Neuroscience Research Australia, Sydney, NSW, Australia. [171]Discipline of Psychiatry and Mental Health, School of Clinical Medicine, Faculty of Medicine and Health, University of New South Wales, Sydney, NSW, Australia. [172]Centro de Biología Molecular Severo Ochoa, Universidad Autónoma de Madrid and CSIC, Madrid, Spain. [173]Department of Psychiatry, Harvard Medical School, Boston, MA, USA. [174]School of Biomedical Science and Pharmacy, University of Newcastle, Newcastle, NSW, Australia. [175]Department of Psychiatry, Taipei Veterans General Hospital, Taipei, Taiwan. [176]Division of Psychiatry, National Yang Ming Chiao Tung University, Taipei, Taiwan. [177]Department of Psychiatry and Human Behavior, School of Medicine, University of California, Irvine, CA, USA. [178]Psychiatry, Psychiatrisches Zentrum Nordbaden, Wiesloch, Germany.

[179]Department of Child and Adolescent Psychiatry/Psychology, Erasmus MC Sophia Children Hospital, Erasmus University, Rotterdam, The Netherlands. [180]Department of Psychology Education and Child Studies, Erasmus School of Social and Behavioral Sciences, Erasmus University Rotterdam, The Netherlands. [181]Department of Research, Innovation and Education, Division of Clinical Neuroscience, Oslo University Hospital, Oslo, Norway. [182]Department of Neurology, Oslo University Hospital, Oslo, Norway. [183]Samsung Genome Institute, Samsung Medical Center, Sungkyunkwan University School of Medicine, Seoul, Republic of Korea. [184]Department of Psychological Medicine, Institute of Psychiatry, Psychology and Neuroscience, King's College London, London, UK. [185]South London and Maudsley NHS Foundation Trust, Bethlem Royal Hospital, Monks Orchard Road, Beckenham, Kent, UK. [186]A list of members and affiliations appears in the Supplementary Note. [187]Department of Clinical Sciences, Psychiatry, Umeå University Medical Faculty, Umeå, Sweden. [188]National Institute of Mental Health, Klecany, Czech Republic. [189]Institute of Environmental Medicine, Karolinska Institutet, Stockholm, Sweden. [190]Department of Psychiatry, University of Münster, Münster, Germany. [191]Department of Psychiatry, Melbourne Medical School, The University of Melbourne, Melbourne, VIC, Australia. [192]The Florey Institute of Neuroscience and Mental Health, The University of Melbourne, Parkville, VIC, Australia. [193]Université Paris Cité, INSERM, Optimisation Thérapeutique en Neuropsychopharmacologie, UMRS-1144, Paris, France. [194]APHP Nord, DMU Neurosciences, GHU Saint Louis-Lariboisière-Fernand Widal, Département de Psychiatrie et de Médecine Addictologique, Paris, France. [195]Psychiatry, University of Pennsylvania, Philadelphia, PA, USA. [196]Center for Statistical Genetics and Department of Biostatistics, University of Michigan, Ann Arbor, MI, USA. [197]University of Queensland, Brisbane, QLD, Australia. [198]Neuropsychiatric Genetics Research Group, Dept of Psychiatry and Trinity Translational Medicine Institute, Trinity College Dublin, Dublin, Ireland. [199]National and Kapodistrian University of Athens, 1st Department of Psychiatry, Eginition Hospital, Athens, Greece. [200]School of Biomedical Sciences, Faculty of Medicine and Health, University of New South Wales, Sydney, NSW, Australia. [201]Department of Human Genetics, University of Chicago, Chicago, IL, USA. [202]Biometric Psychiatric Genetics Research Unit, Alexandru Obregia Clinical Psychiatric Hospital, Bucharest, Romania. [203]Department of Psychiatry, Department of Psychiatric Genetics, Poznan University of Medical Sciences, Poznan, Poland. [204]School of Medicine and Public Health, University of Newcastle, Newcastle, NSW, Australia. [205]Department of Medical Biochemistry and Biophysics, Karolinska Institutet, Stockholm, Sweden. [206]HUNT Research Center, Department of Public Health and Nursing, Faculty of Medicine and Health Sciences, Norwegian University of Science and Technology, Trondheim, Norway. [207]Department of Public Health and Institute of Epidemiology and Preventive Medicine, College of Public Health, National Taiwan University, Taipei, Taiwan. [208]Neuroscience Therapeutic Area, Janssen Research and Development, LLC, Titusville, NJ, USA. [209]JRD Data Science, Janssen Research and Development, LLC, Titusville, NJ, USA. [210]Cancer Epidemiology and Prevention, M. Sklodowska-Curie National Research Institute of Oncology, Warsaw, Poland. [211]SA MRC Unit on Risk and Resilience in Mental Disorders, Dept of Psychiatry, Stellenbosch University, Stellenbosch, South Africa. [212]University of Newcastle, Newcastle, NSW, Australia. [213]Department of Psychiatry, Amsterdam University Medical Center, Amsterdam, The Netherlands. [214]Department of Psychiatry and Neuropsychology, School for Mental Health and Neuroscience, Maastricht University Medical Center, Maastricht, The Netherlands. [215]School of Psychology, The University of Queensland, Brisbane, QLD, Australia. [216]Department of Psychiatry and Genetics Institute, University of Florida, Gainesville, FL, USA. [217]Research Institute, Lindner Center of HOPE,

Mason, OH, USA. [218]School of Psychology and Faculty of Medicine, The University of Queensland, Brisbane, QLD, Australia. [219]School of Psychology and Counselling, Queensland University of Technology, Brisbane, QLD, Australia. [220]Division of Mental Health and Addiction, University of Oslo, Institute of Clinical Medicine, Oslo, Norway. [221]Department of Mental Health, Faculty of Medicine and Health Sciences, Norwegian University of Science and Technology (NTNU), Trondheim, Norway. [222]Psychiatry, St Olavs University Hospital, Trondheim, Norway. [223]Psychosis Research Unit, Aarhus University Hospital - Psychiatry, Risskov, Denmark. [224]NCRR and CIRRAU, Aarhus BSS, Aarhus University, Aarhus, Denmark. [225]Munich Cluster for Systems Neurology (SyNergy), Munich, Germany. [226]University of Liverpool, Liverpool, UK. [227]HudsonAlpha Institute for Biotechnology, Huntsville, AL, USA. [228]Medical and Population Genetics, Broad Institute, Cambridge, MA, USA. [229]Mental Health Services in the Capital Region of Denmark, Mental Health Center Copenhagen, University of Copenhagen, Copenhagen, Denmark. [230]Psychiatry, Indiana University School of Medicine, Indianapolis, IN, USA. [231]Division of Psychiatry, Haukeland Universitetssjukehus, Bergen, Norway. [232]Faculty of Medicine and Dentistry, University of Bergen, Bergen, Norway. [233]Department of Clinical Neuroscience and Center for Molecular Medicine, Karolinska Institutet at Karolinska University Hospital, Solna, Sweden. [234]Human Genetics and Computational Biomedicine, Pfizer Global Research and Development, Groton, CT, USA. [235]Melbourne Neuropsychiatry Centre, Department of Psychiatry, The University of Melbourne, VIC, Australia. [236]Monash Institute of Pharmaceutical Sciences (MIPS), Monash University, Parkville, VIC, Australia. [237]Rutgers Health, Rutgers University, Piscataway, New Jersey, USA. [238]University of Patras, School of Health Sciences, Department of Pharmacy, Laboratory of Pharmacogenomics and Individualized Therapy, Patras, Greece. [239]United Arab Emirates University, College of Medicine and Health Sciences, Department of Genetics and Genomics, Al-Ain, United Arab Emirates. [240]United Arab Emirates University, Zayed Center for Health Sciences, Al-Ain, United Arab Emirates. [241]Erasmus University Medical Center Rotterdam, Faculty of Medicine and Health Sciences, Department of Pathology, Clinical Bioinformatics Unit, Rotterdam, The Netherlands. [242]Department of Neurology and Neurosurgery, McGill University, Faculty of Medicine, Montreal, QC, Canada. [243]Montreal Neurological Institute and Hospital, McGill University, Montréal, QC, Canada. [244]Center for Precision Medicine and Translational Therapeutics, James J. Peters VA Medical Center, Bronx, NY, USA. [245]Centre for Brain and Mental Health Research, The University of Newcastle, Newcastle, NSW, Australia. [246]Hunter Medical Research Institute, New Lambtion Heights, NSW, Australia. [247]Department of Psychiatry and Psychotherapy, University Medical Center Göttingen, Göttingen, Germany. [248]Department of Psychiatry and Behavioral Sciences, SUNY Upstate Medical University, Syracuse, NY, USA. [249]The School of Biomedical Sciences and Pharmacy, Faculty of Medicine, Health and Wellbeing, University of Newcastle, Newcastle, NSW, Australia. [250]Cancer Detection and Therapies Program, Hunter Medical Research Institute, University of Newcastle, Newcastle, NSW, Australia. [251]Department of Medicine and Surgery, Kore University of Enna, Enna, Italy. [252]Department of Biomedical and Neuromotor Sciences, University of Bologna, Bologna, Italy. [253]Oasi Research Institute-IRCCS, Troina, Italy. [254]Department of Psychiatry, Massachusetts General Hospital, Boston, MA, USA. [255]Psychiatric and Neurodevelopmental Genetics Unit (PNGU), Massachusetts General Hospital, Boston, MA, USA. [256]Faculty of Medicine, University of Iceland, Reykjavik, Iceland. [257]Department of Psychiatry, Hospital Namsos, Namsos, Norway. [258]Department of Neuroscience, Norges Teknisk Naturvitenskapelige Universitet Fakultet for naturvitenskap og teknologi, Trondheim, Norway. [259]Hector Institute for Artificial Intelligence in Psychiatry, Central Institute of Mental

Health, Medical Faculty Mannheim, Heidelberg University, Mannheim, Germany. [260]Department of Genetics, University of North Carolina at Chapel Hill, Chapel Hill, NC, USA. [261]Department of Psychiatry, University of North Carolina at Chapel Hill, Chapel Hill, NC, USA. [262]Department of Psychiatry, McGill University, Montreal, QC, Canada. [263]Dept of Psychiatry, Sankt Olavs Hospital Universitetssykehuset i Trondheim, Trondheim, Norway. [264]Clinical Institute of Neuroscience, Hospital Clinic, University of Barcelona, IDIBAPS, CIBERSAM, Barcelona, Spain. [265]Department of Psychology, Emory University, Atlanta, GA, USA. [266]Department of Neuroscience, SUNY Upstate Medical University, Syracuse, NY, USA. [267]Institute of Biological Psychiatry, Mental Health Services, Copenhagen University Hospital, Copenhagen, Denmark. [268]Department of Clinical Medicine, University of Copenhagen, Copenhagen, Denmark. [269]Center for GeoGenetics, GLOBE Institute, University of Copenhagen, Copenhagen, Denmark. [270]Biochemistry and Molecular Biology, Indiana University School of Medicine, Indianapolis, IN, USA. [271]Department of Medical and Molecular Genetics, Indiana University, Indianapolis, IN, USA.
